# A Combined Semi-Supervised Deep Learning Method for Oil Leak Detection in Pipelines Using IIoT at the Edge

**DOI:** 10.3390/s22114105

**Published:** 2022-05-28

**Authors:** Christos Spandonidis, Panayiotis Theodoropoulos, Fotis Giannopoulos

**Affiliations:** Prisma Electronics SA, Leof. Poseidonos 42, 17675 Kallithea, Greece; panagiotis.theodoropoulos@prismael.com (P.T.); fotis.giannopoulos@prismael.com (F.G.)

**Keywords:** leakage detection, oil pipeline, deep learning, CNN classifier, LSTM autoencoders, edge computing

## Abstract

Pipelines are integral components for storing and transporting liquid and gaseous petroleum products. Despite being durable structures, ruptures can still occur, resulting not only in financial losses and energy waste but, most importantly, in immeasurable environmental disasters and possibly in human casualties. The objective of the ESTHISIS project is the development of a low-cost and efficient wireless sensor system for the instantaneous detection of leaks in metallic pipeline networks transporting liquid and gaseous petroleum products in a noisy industrial environment. The implemented methodology is based on processing the spectrum of vibration signals appearing in the pipeline walls due to a leakage effect and aims to minimize interference in the piping system. It is intended to use low frequencies to detect and characterize leakage to increase the range of sensors and thus reduce cost. In the current work, the smart sensor system developed for signal acquisition and data analysis is briefly described. For this matter, two leakage detection methodologies are implemented. A 2D-Convolutional Neural Network (CNN) model undertakes supervised classification in spectrograms extracted by the signals acquired by the accelerometers mounted on the pipeline wall. This approach allows us to supplant large-signal datasets with a more memory-efficient alternative to storing static images. Second, Long Short-Term Memory Autoencoders (LSTM AE) are employed, receiving signals from the accelerometers, and providing an unsupervised leakage detection solution.

## 1. Introduction

During recent decades, pipeline networks have been considered among the safest and most economical methods for transporting and storing oil and gas products [1]. In fact, pipeline infrastructure is critical for worldwide economic growth. Multiple investments in hydrocarbons and petrochemical facilities are materialized thanks to the steady and reliable supply of feedstocks provided by pipeline infrastructure [2]. For example, it has been estimated that, in 2015, crude oil pipelines generated approximately 200,000 jobs, accumulating over $21.8 billion in Gross Domestic Product [3]. Consequently, oil piping installations worldwide have been rapidly expanding to satisfy the ever-increasing energy needs of the population, intricating the topological complexity of the pipeline network, perplexing its supervision and assessment of its safety [4]. Additionally, this breadth of pipeline usage inherently aggrandizes the probability of structural defects due to erosion over time, fracture propagation, human factors, environmental factors, and other causes [5,6,7]. Leak detection in pipelines has been a prevalent issue for several decades. Pipeline leaks from sources such as small cracks and pinholes are termed chronic leaks, as they have the potential of going unnoticed for a long period of time, causing irreversible damage [8]. Even seemingly small defects scale up fast to unfathomable magnitude. For instance, on 2 March 2006, a spill of about 1 million liters of oil occurred over around five days in the area known as Alaska’s North Slope because a quarter-inch hole corroded in a pipeline [9]. Therefore, ensuring the apt functioning of these pipelines is imperative to avert excessive financial losses due to the interruption of oil and gas supply and, most importantly, to eliminate any potential threat to human lives and the ensuing detrimental aftermath on the environment.

Several conventional approaches undertake the recognition of defects on pipelines by analyzing their vibration response characteristics, using digital signal processing techniques, such as fast Fourier transform [10] and the wavelet transforms [11]. More recently, following the fourth Industrial revolution, Machine Learning data-driven approaches have gained popularity due to their high accuracy compared to other conventional methods and their efficient implementation due to recent advancements in tensor multiplication dedicated GPUs. In this vein, Deep Learning is widely employed to perform leakage detection in pipeline systems, aiming to leverage their efficacy in identifying even relatively small leakage diameters [12], processing data either in the time domain [13,14] or in the frequency domain [15]. Autoencoders are also a class of neural networks whose attribute of being trained on unlabeled data and distinguishing potential digression from the nominal state becomes very beneficial for detecting faulty conditions in the pipelines [16,17]. Convolutional Neural Networks (CNN) are utilized to perform feature extraction and learn through a series of filters to identify salient features in the data. Subsequently, these feature maps are fed to Multi-Layer Perceptrons (MLPs) [18,19,20] or Support Vector Machines (SVM) [21] to determine the operational state reflected by the initial signal. Although feature extraction is integral for classification data-driven methods [22], their main limitation is that they require a high computational cost. Post-processing analysis is performed on historical data in the cloud, and a need for the storage of a high volume of data makes both the training and execution time of the models inefficient.

To address these issues, the “ESTHISIS” project [23] aims to employ edge computing to apply DL techniques in real-time and detect leakages in oil and gas pipelines. In this framework, our novelty lies in the emphasis on providing Situational Awareness of the oil and gas pipelines to stakeholders through the harmonious integration of our wireless sensor networking to enhance the operational capacity of oil and gas pipelines. In our previous study [24], two DL methodologies were presented in two different experimental setups and compared with their efficiency in detecting leakages in pipelines. The first method entails a supervised approach based on transforming the data to the time-frequency domain, creating spectrograms from the acquired sensors data, and using a 2D-Convolution Neural Network to characterize whether the pipeline is healthy or not. The second method is an unsupervised approach employing Long Short-Term Memory Autoencoders (LSTM AE) trained to reconstruct signals from healthy channels. The focal point of the current work is to merge these techniques efficiently, leverage the benefits yielded by each method, and present a comprehensive leakage detection scheme that can run on the edge to provide efficiency and scalability.

The main innovation of our work is the development of an edge methodology capable of running DL applications in a scalable manner for real-time analytics and providing accurate estimations for leakage detection. Our modeling entails a hybrid approach where the components of our previous analysis offer the capability of training the model simply by utilizing signals to correspond to the nominal healthy state (LSTM AE) and also present the benefit of converting long time series into low-resolution static images, which is a more memory-efficient solution (2D-CNN). Emphasis has been placed on the methodology’s validation through an experimental pipeline network during in-field testing. The dataset acquired from these trials is utilized for training and optimizing an instance of our proposed combined approach, which shall be stored on the edge. Subsequently, this instance shall be evaluated by undertaking real-time analytics to detect leakages in actual operating pipelines. In this context, parametric tests were performed to verify the model accuracy and efficiency in the actual environment within oil premises.

The rest of the paper is structured as follows: In Section 2, a description of the processing system responsible for the data acquisition is provided. In Section 3, the methodology behind our detection scheme is delineated. Section 4 and Section 5 present the results from the experimental field-testing phase and pilot testing. Finally, the conclusion from the performance of each model is summarized.

## 2. Sensor Network and Data Acquisition

### 2.1. System Architecture

**Figure 1:**
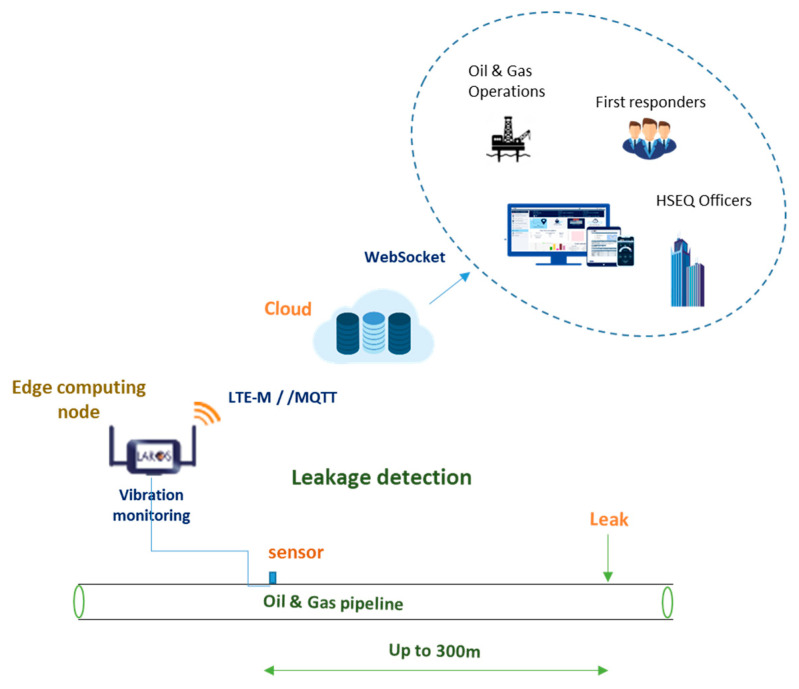
Leak detection mode architecture. Systems mounted on the pipeline are depicted in green, while blue for cloud-based services.

The general architecture of the ESTHISIS system [25] is presented in Figure 1. The platform architecture is based on PrismaSense™ technology [26,27,28] which was further enhanced with edge computing capacity. The ESTHISIS platform system serves two operational modes: (a) leak detection and (b) leak localization mode. The leak detection mode is the default mode of operation and is the focus of the current work.

As shown, an intelligent node is placed along a pipeline and collects data from vibration sensors. While a series of similar nodes are mounted on the pipeline with a distance of up to 300 m between them, in this mode, each node acts as a stand-alone system that collects and processes the data to detect any leakage, based on the leak detection method mentioned in Section 3. Upon the detection of leakage, the node transmits a dedicated message to the cloud, using a Narrow Band IoT ((NB-IoT) communication link for visualization, trigger, and further analysis. Different communication protocols (e.g., satellite communications) are considered for areas with no NB-IoT coverage. The mode is characterized by running on-demand and at periodic intervals. This type of data acquisition with advanced signal processing algorithms enables the device to identify leakage in an oil and gas pipeline. The ability to operate the system on demand enables the user to inspect the system remotely and on-demand in real-time.

### 2.2. Hardware

**Figure 2:**
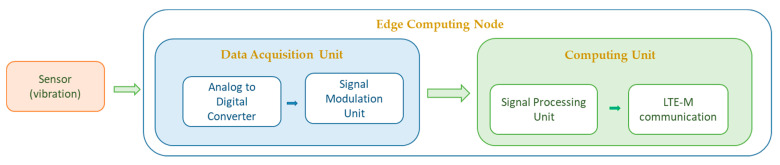
Node design architecture.

Figure 2 illustrates the design architecture of the nodes. As shown, signals emitted from the pipeline are collected by the accelerometers at each node and are processed. These signals are amplified, filtered, and then digitized by an ADC. The data are collected by the Microcontroller and transferred to the Microprocessor until their processing. The data are processed locally at each node level.

While the difference between the primary and the secondary node is that the latter sends its data to the former via the communication unit, the two nodes have the same architecture since each node can act both as primary and secondary. This architecture enables the easy scalability of the system since the addition of more nodes is straightforward: each node acts as a primary to the node in its right and secondary for the node to its left. The node’s main hardware components are described in this section. Its technical characteristics are presented in Table 1.

**Figure 3:**
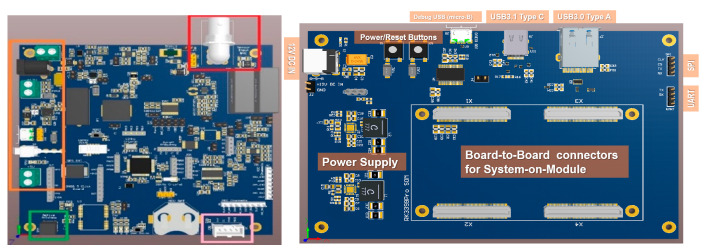
Sensor Data Receiving (**left**) and Data Processing (**right**) Interfaces.

The Sensor Data Receiving Interface (Figure 3, left) is a PCB designed to receive data from up to four accelerometers, synchronize them using a PPS (Pulse Per Second) signal, and transmit them to the Data Processing Interface. The main components are the Analog to Digital Converter (ADS8688 [29]) that receives the analog sensors’ data and converts them to digital data, the GPS Unit that produces the timestamp for each data measurement, and the PPS signal for synchronization with great accuracy and the Microcontroller that sends the digitized data to the Data Processing Interface. The GPS Unit contributes so that the two nodes receive fully-synchronized data measurements. The suitable GPS Unit for the project is the NEO-M8N by Ublox [30]. Moreover, the Microcontroller selected is the ESP32 Wrover module by Espressif [31].

The Data Receiving Interface is connected via micro-B USB with the Data Processing Interface (Figure 3, right) for power supply. Furthermore, there is the option of power supply by a +12 V jack. Regarding the Data Processing Interface, a PCB was designed and manufactured for processing the data received from the accelerometers. The main component is the RK3399Pro System-On-Module, which consists of a dual-core ARM Cortex-A72 and a quad-core ARM Cortex-A53 microprocessors. The Data Processing Interface is supplied by +12 VDC/2 A.

### 2.3. Software

**Figure 4:**
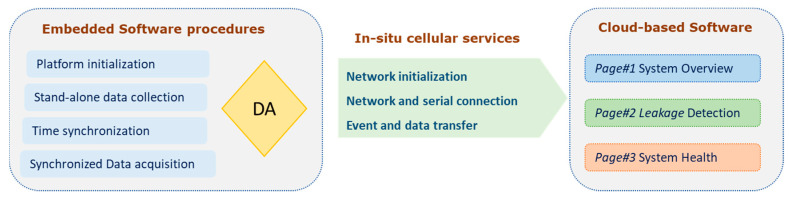
ESTHISIS Software procedures.

The platform involves a series of embedded software procedures responsible for the Data Acquisition (DA) to feed the embedded Artificial Intelligence algorithms through an LTE-M cellular network. A web application is hosted on the Central Unit and displays information about the location and the size of leakage to the users as soon as it occurs. The web application also provides monitoring of the nodes’ operating conditions, as well as statistical information related to their operation. The software procedures are summarized in Figure 4 and are further described in the following sections.

The web application developed for the ESTHISIS project aims to inform its users about leakages along the pipelines on which nodes have been placed. It also allows the monitoring of the pipeline network condition in sections, the monitoring of the condition of the nodes, and the display of information about the whole system.

## 3. Method Description

### 3.1. Methodology Components

In our problem, the data consisted of a univariate time series, with only variables stemming from the acoustic signals from the pipeline wall. We built an LSTM AE on this univariate time series to perform rare-event recognition.

**Figure 5:**
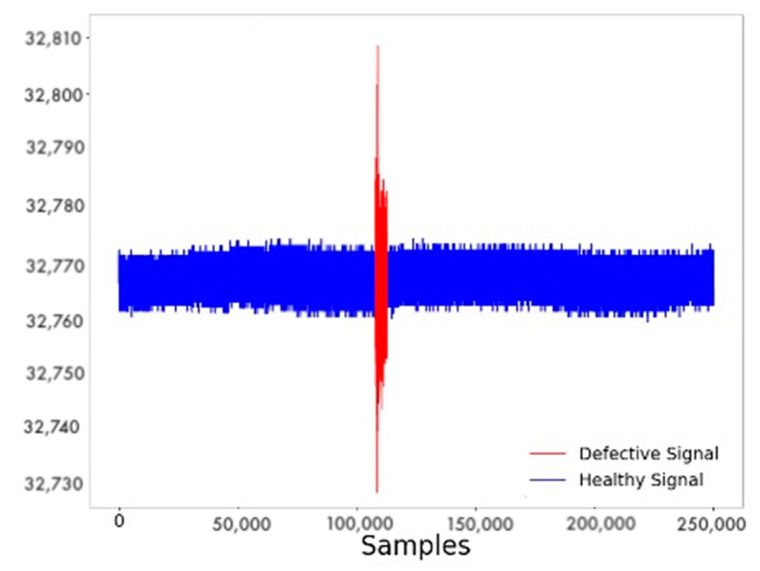
Example of leakage detection using LSTM AE.

In our study cases, the LSTM autoencoders were trained only on healthy signals. The objective of these networks was to reduce the divergence between the input and the reconstructed input at the model’s output. In its attempt to satisfy this objective, the model achieved great fidelity in the reconstruction of the input. After the training, the model inferred the state of the validation set again, consisting of different healthy signals from the training set. Subsequently, through an extensive trial and error approach, it was concluded that the mean of the reconstruction error added to its standard deviation multiplied by 6 approximately equates to the maximum reconstruction error observed in the validation set multiplied by a safety factor of 1.2. These two almost equal values serve as the leakage threshold. Lastly, a range of 1.1–1.5 times the maximum reconstruction error was considered a reasonable range for the selection of the safety factor, depending on the level of conservativeness in the generated estimations. In our approach, the latter value was selected due to its simpler implementation. In essence, since the autoencoders have been trained to reproduce healthily, their reconstruction error was expected to be below this threshold. Therefore, exceeding the leakage threshold signifies leakage in the pipeline. Figure 5 illustrates a characteristic example.

**Figure 6:**
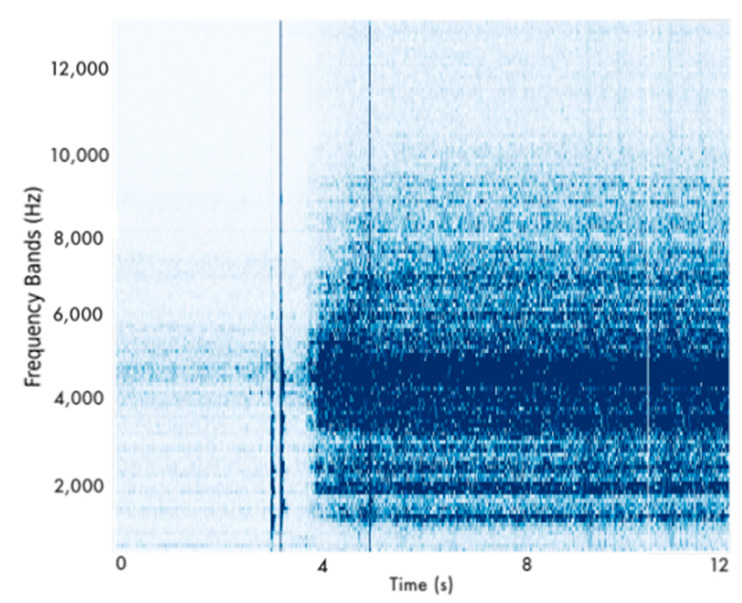
Example of spectrogram received by the CNN classifiers.

Subsequently, if leakage was detected, the generation of spectrograms ensued. The conversion of the signals to static images was considered. Due to the high-frequency dataset, we concluded to generate spectrograms to reflect the operational state of the monitored pipelines. This method offered a more memory-efficient alternative to representing lengthy signals in static images. Explicitly, a file representing the time series of 10 s of the data occupied more than 10 MB of memory. Conversely, the spectrogram image of the same signal of resolution (256 × 256) occupied less than 300 KB. The classification of the spectrograms was undertaken through Convolutional Neural Networks (CNNs). Figure 6 illustrates an example of a spectrogram received by the CNN classifiers. CNNs are neural networks that demonstrate excellent capabilities in pattern recognition. Their capacity to extract salient features without requiring prior domain expertise prompts researchers and developers to approach complex pattern recognition tasks.

#### Combined LSTM AE—2D CNN Approach

In our previous study, the LSTM AE and 2D-CNNs models were introduced and verified as effective data-driven models potent at identifying patterns in the data signifying leakage. It was evinced that LSTM AE models were capable of timely detecting leakage in the pipelines; however, they displayed limitations in continuously labeling the operational state of a defective pipeline as defective in small leakages. Additionally, the efficacy of 2D-CNNs was evaluated in classifying spectrograms derived from the signals acquired from the pipeline walls. Given that the instantaneous detection was satisfactorily fulfilled by the LSTM models, it was decided to omit the time points where efflux occurs from these spectrograms to examine the efficacy of these classifiers to detect leakage in case the occurrence of the rupture in the pipeline is not recorded; hence the spectrograms were appropriately cropped. It was concluded that these two models could be complementary components of one comprehensive methodology. The initial stage concerns the LSTM AE monitoring the response from the spectrograms mounted on the pipeline wall. Subsequently, if an abnormality was detected following the mechanism that shall be described in Section 3.3, the generation of the spectrograms was initiated for the continuous labeling of the monitored pipeline’s state as defective and the storing of the operational information in a more efficient image format.

### 3.2. Model Training

#### 3.2.1. LSTM AE

**Figure 7:**
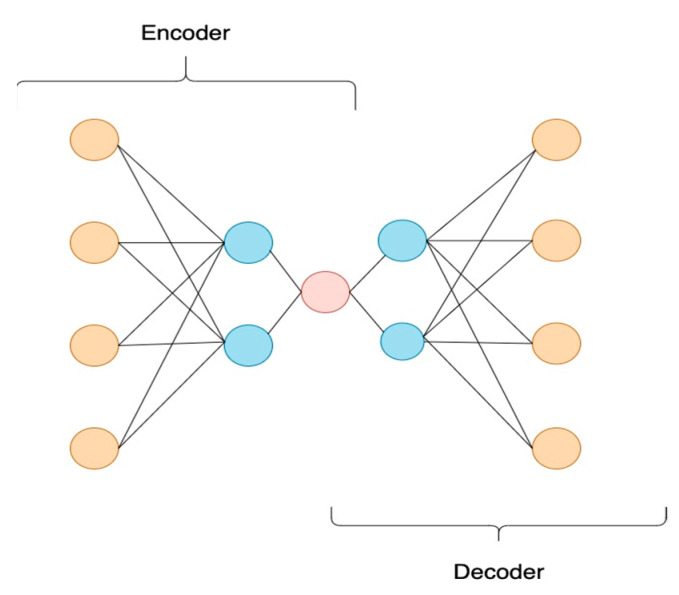
Demonstration of the architecture of a typical Autoencoder.

In its simplest form, an autoencoder is a class of neural networks used for the efficient reconstruction of unlabeled data. The autoencoder learns a representation for a given dataset by training the network to ignore insignificant parts of the data, such as noise. In anomaly detection, we learn the pattern of a normal process. Anything that does not follow this pattern is classified as an anomaly. An autoencoder has two main parts, as illustrated in Figure 7.

The first part is the encoder that maps the input into the latent representation h, and a decoder that maps the information of the latent space to a reconstruction of the input.

In the simplest case, given one hidden layer, the encoder stage of an autoencoder takes the $x\;\in \;{\mathbb{R}}^{d}$ and maps it to $h\;\in \;{\mathbb{R}}^{p}$. Utilizing this information, we can express latent space ***h*** as follows:

(1) h=σ(Wx+b) 

This image ***h*** is often referred to as a latent representation or latent space. *σ* is an activation function such as the sigmoid or the ReLU activation function. ***W*** is the weight matrix, and ***b*** is the bias vector, usually initialized randomly, but as the training progresses, they are updated incrementally through backpropagation. Subsequently, the decoder receives the latent representation ***h*** and ultimately tries to reconstruct the encoder’s input. In other words, the decoder attempts to map the latent representation ***h*** to the reconstruction $x^{\prime }$. Under our previous notation, this operation was formulated as follows:

(2) $$x^{\prime }=\sigma^{\prime }\left(W^{\prime }h+b^{\prime }\right)\;$$

where *σ* is again an activation function, and ***W*** and ***b*** are the weight matrix and the bias vector, respectively. We underline that $x^{\prime },\;\sigma^{\prime },\;b^{\prime }$ are disparate from their encoder counterparts.

Ultimately, the autoencoders were trained to minimize the loss function, also referred to as reconstruction errors. For instance, an example of a reconstruction loss may be the Mean Square Error:

(3) $$L\left(x,\;x^{\prime }\right)=\frac{1}{n}\overset{n}{\underset{i=1}{\sum }}{\left(x-x^{\prime }\right)}^{2}=\frac{1}{n}\overset{n}{\underset{i=1}{\sum }}{\left(x-\sigma^{\prime }\left(W^{\prime }\left(\sigma \left(Wx+b\right)\right)\right)+b^{\prime }\right)}^{2}\;$$

**Figure 8:**
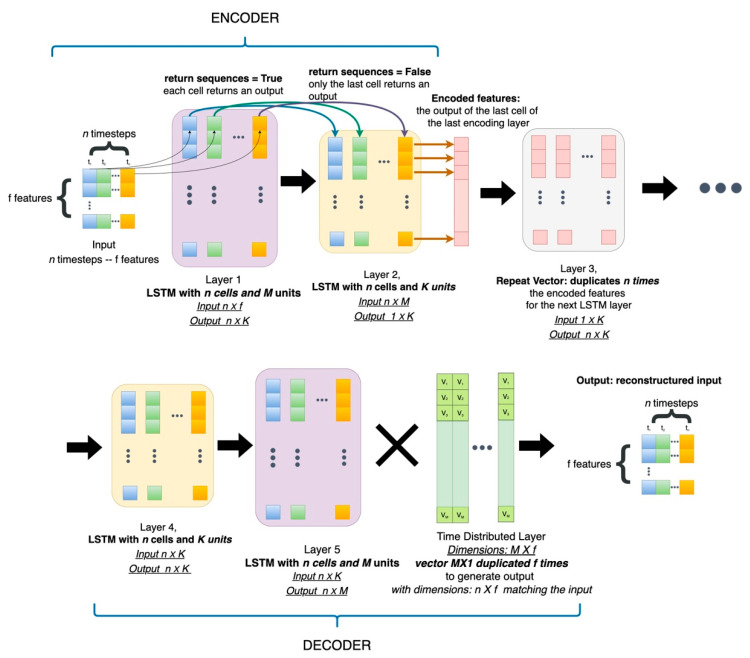
The architecture of LSTM AutoEncoders.

In our study case, due to the sequential trait of the signals, we developed LSTM AE, which were suitable to process the time series thanks to their feedback connections, as illustrated in Figure 8. An LSTM AE was developed on this univariate time series to perform rare-event classification. Given a lookback window dictating the extent of the time series patch received by the network, the information flow is visualized in Figure 8. It was observed that the LSTM network received a 2D array with dimensions *n* × *f* as input at each timestep. The dimensions of this array corresponded to the n prior timesteps the network considered at each input and the f features comprising the dataset. The LSTM layers consisted of as many cells as the number of time points the network looks back at each time *t*. In a sequence of LSTM layers, every cell of the preceding layer generates an output to construct the 2D array the following layer requires.

To generate the reconstructed input, the output of the last LSTM layer must be multiplied by a 2D array. Essentially, this array is a vector of length equal to the number of units in each cell of the last LSTM layer, repeated f times, namely as many times as the number of features in the input. Ultimately, the goal of these networks was to minimize the divergence between the input and the reconstruction at the model’s output by minimizing the reconstruction error as defined in (3). The satisfaction of this stipulation ensured the fidelity of the reconstruction to the ground truth data.

The advantage of this method was that there was no need to label the samples before training. More specifically, LSTM AE provided an unsupervised inspection. Our developed model was trained based on the signals from the experimental setup in Kalochori, where no leakages were induced. The objective during the training phase was to minimize the reconstruction error. Therefore, it ensured that at the end of the training, the autoencoder was capable of reconstructing healthy signals with excellent fidelity. After the model’s training, another set of signals separated from the training set, likewise consisting of only healthy time series stemming from the Kalochori setup, was passed through the autoencoder to audit the model’s performance. The maximum reconstruction error observed in the validation set multiplied by a safety factor served as the leakage threshold.

In essence, since the autoencoders regenerated the healthy signals with great precision, their reconstruction error was less than the threshold. Contrarily, if the reconstruction error was significantly augmented, exceeding this threshold during the testing phase, this event signified leakage in the pipeline. In this manner, the LSTM model was trained from the experimental setup to monitor the pipelines in the actual working environment.

#### 3.2.2. Convolutional Neural Networks

The next component of our leakage detection pipeline was the CNN classifier. This model undertook the supervised classification of the generated spectrograms. This network was trained based on the spectrograms generated from the validation dataset stemming from the validation trials run in the experimental pipeline setup in Kalochori. This dataset consisted of equally healthy and defective samples to train the classifier adequately to identify these states.

The operation of convolution enabled these networks to learn efficiently and automatically detect significant features in the images without requiring human intervention. This implied that CNNs independently performed the arduous task of feature engineering, relieving engineers and researchers of that burden. Explicitly, the developed classifier was trained to identify features in the input images and determine the state of the pipeline. This operation was essentially a linear element-wise multiplication (dot product) between the small array called the kernel of dimensions Na × Nb gliding through the input tensor of the layer and the elements of this tensor. This process with multiple kernels allowed the networks to recognize diverse patterns in the input images. Summing the fragments generated by the dot product between the filter and the corresponding portion of the input tensor, its auditing yielded the value of the output tensor in the respective position. Convolution on the image, namely on a 2D plane, with a resolution of H × W and Nc color channels, takes place as follows:

(4) $$F\left(x,y,k\right)=K{}^{\circ}S\left(x,y,k\right)=\overset{\mathrm{Na}}{\underset{i=1}{\sum }}\overset{\mathrm{Nb}}{\underset{j=1}{\sum }}\overset{\mathrm{Nc}}{\underset{k=1}{\sum }}K\left(i,j,k\right)S\left(x+i-1,y+j-1,k\;\right)\;$$

The conclusion of this pattern of alternating convolutional and pooling layers leads to the flattening of the final output tensor into a vector, which constitutes the input of a traditional FC Network succeeding the configuration of convolution and pooling layers. The output of a node *j* at the *ℓ*th layer of the Fully Connected Network can be expressed as follows:

(5) $$z_{j}^{\left[l\right]}=W^{\left[l\right]T}\cdot a^{\left[L-1\right]}+b=\overset{n^{\left[l-1\right]}}{\underset{k=1}{\sum }}w_{jk}^{\left[l\right]}a_{k}^{\left[l-1\right]}+b_{j}^{\left[l-1\right]}\;$$

(6) $$\to \;a_{j}^{\left[l\right]}=f\left(z_{j}^{\left[l\right]}\right)$$

where f(x)=ReLU(x)=max(0,x) and $n^{\left[l-1\right]}$: the number of nodes at the previous layer.

Based on the feature extraction by the convolution layer and the forward propagation of the information in the FC layers, the nodes of the last layer of the FC Network and, by extension, of the whole CNN, classify the state of the piping network reflected by the sample image. The output of the last layer converts the estimations of the network into a probability distribution over the predicted classes through the SoftMax activation function; hence the summation of all outputs adds to 1.

(7) $$\;a^{\left[L\right]}=Softmax\left(W^{T}\cdot a^{\left[L-1\right]}+b\right)=\frac{e^{z_{i}^{\left[L\right]}}}{\sum_{i=1}^{M}e^{z_{i}^{\left[L\right]}}}$$

Loss: In neural networks, the loss function quantifies the digression between the predicted values and the ground truth labels assigned to the samples of the dataset. Therefore, the minimization of the loss function constitutes the primary objective during the training of the networks. The Categorical Cross-Entropy (CCE) loss function was employed in our classification task. Assuming a dataset consisting of N observations, the vector containing the ground truth labels of the samples was denoted as $y=[y_{1},\;y_{2},$ …, $y_{n}]$, each assigned to 1 of a total of M labels. Additionally, following the notation previously used, the predicted values are the output values of the last layer, thus being denoted as $a^{\left[L\right]}=\left[\;a_{1}^{\left[L\right]},\;a_{2}^{\left[L\right]},\;\ldots ,\;a_{n}^{\left[L\right]}\right]$. Therefore, the expression for the CCE loss function can be written as demonstrated in Equation (2):

(8) $$E=CCE\left(y,a^{\left[L\right]}\right)=-\overset{M}{\underset{i=1}{\sum }}y_{i}\log\left(a_{i}^{\left[L\right]}\right)$$

Thus substituting (7) into (8), the following expression is obtained:

(9) $$E=-\overset{M}{\underset{i=1}{\sum }}y_{i}\log\left(\frac{e^{z_{i}^{\left[L\right]}}}{\sum_{i=1}^{M}e^{z_{i}^{\left[L\right]}}}\right)$$

Backpropagation: After the completion of the forward pass, the backpropagation succeeds, in the context of which the learnable parameters of the network are updated, attempting to accomplish convergence of the output predictions and the actual values of the samples, hence minimizing the loss function. For this matter, the calculation of the gradient of the loss function of each learnable parameter took place, and it was subsequently used to update the respective parameter by an arbitrary step, determined by the learning rate, which is a hyperparameter. This process can be formulated as follows:

(10) $$p=p-a\;\frac{\partial L}{\partial p}\;$$

where *p* represents any learnable parameter of the model and α represents the selected learning rate.

### 3.3. Detection Mechanism

The detection scheme began with the identification of the leakage through the LSTM AE component. Our decision system for labeling a signal as defective was the following: if out of the 25,000 measurements collected in 1 s, the number of observations above the leakage threshold is larger than 10,000, the system was flagged as defective. The number “10,000” was arbitrarily selected, and it manifests our emphasis on averting false negatives, namely indications where the system does not identify potential system defectiveness, preventing the occurrence of false positives in the event of erroneous measurement, which would lead the autoencoder to exceed the reconstruction threshold. Denoting with 1 and 0 the Boolean variables True and False, respectively, we formulate our decision process in Equation (15).

(11) $$Possible\;Leakage=\{\begin{array}{c} 1\;if\;Error>Threshold\\ \;0\;if\;Error<Threshold \end{array}$$

(12) $$Leakage=\{\begin{array}{c} 1,\;if\;\overset{t+25,000}{\underset{i=t}{\sum }}Possible\;Leakage_{i}>10,000\\ 0,\;if\overset{t+25,000}{\underset{i=t}{\sum }}Possible\;Leakage_{i}<10,000 \end{array}$$

**Figure 9:**
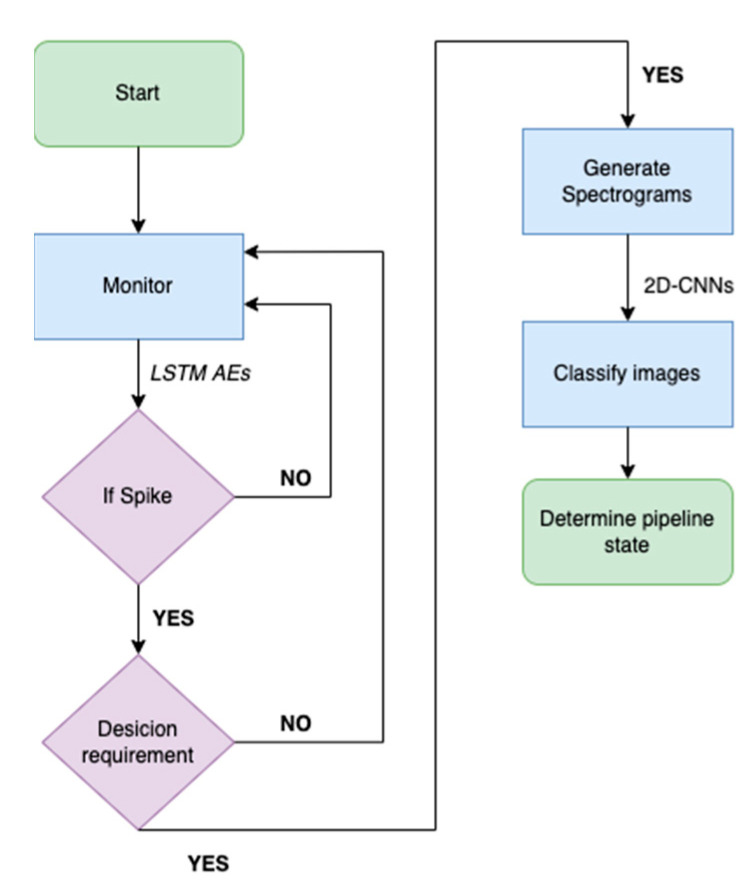
Proposed methodology flowchart.

Second, when the above decision requirement was satisfied and, thus, detected a leakage, it initiated the CNN-2D classification. Where the signal from the pipeline wall was converted into a spectrogram with a rolling 20-s window, namely at each time point, the last 20 s of the signals were transferred into the time-frequency domain, generating a spectrogram. The flowchart in Figure 9 illustrates the operations and controls taking place in our models when monitoring a given signal.

## 4. Method Validation

The first set of experiments was undertaken in an experimental setting, intending to verify the applicability of the proposed methodology. For this matter, the experimental setup pipeline network set up for the ESTHISIS project in Kalochori, Thessaloniki, Greece, hosted this round of experiments. This dataset is utilized for the training and obtaining the optimized instances of the network that shall be used to undertake leakage detection in the subsequent phase of our methodology testing in an actual working environment, as presented in Section 5.

For the initial setup and after any change that altered any significant geometrical parameter of the format, such as the distance between the two sensors, the following process was followed: The water pressure was set to a pre-defined value and remained unaltered throughout the experiments, as it was regularly monitored, and water was added when needed to maintain a steady water pressure inside the pipeline. The two nodes are fully aligned on top of the pipeline wall in order to minimize uncertainties, and the induced leakage is 90° perpendicular to their plane due to mounting limitations posed by the environment. This configuration was selected in order to match the conditions that shall be met in the oil refinery premises during our method verification phase. An initial series of consecutive recordings without any leaks was taken to record a reference vibration signal for the channel. These recordings had a 10-min duration in total. Subsequently, a series of short tests of approximately 10 s each were carried out. During each trial, one of the faucets was turned on to emulate a leakage of a specific diameter. While the sampling rate can be defined by the user in our case, each node sampled the sensors’ analog signals with a sampling frequency of 25 KHz. Each run produced a sample that shall be received by the network and corresponds to 250,000-time steps, given the 10-s duration of each test and the 25 KHz sampling rate.

During the first day of the field tests in Kalochori, the majority of the tests were carried out without water flowing inside the pipeline, whereas most of the experiments carried out during the second day involved water flowing inside the channel. The other parameters that changed during the field tests were the distance between the sensors placed on the pipeline, the distance between the leakage and the sensors, and the diameter of the leakage ranging from 1 mm to 7 mm, i.e., the diameter of the faucet that was turned on each time. According to the standard test practice, each testing procedure was repeated 12 times. Table 2 presents the number of available signals along with their properties.

Additionally, Table 2 indicates the number of samples corresponding to each subset. Lastly, Table 3 and Table 4 tabulate the selected architecture and hyperparameter configuration of the trained and optimized models that shall be employed for the anomaly detection tests in the real environment.

## 5. Verification in a Real Environment

The optimized model that occurred from the experimental tests in Kalochori was used to monitor the pipelines in this setup and detect anomalies that imply leakage in the actual operating environment in oil refinery premises. The pipeline network for the field tests in a real environment was similar to the one described in Section 4 for the field tests in Kalochori. The main difference between these two lies in the considerable ambient noise extant in the measurements that stem from other ongoing procedures taking place at the facilities, instigating external noise entering the monitored system. Additional differences between the two setups were the pipeline diameter since the available valves generated leakages of 5 mm and 13 mm.

The challenge of this analysis lies in the considerable ambient noise interfering with the measurements from other procedures taking place at the facilities. Similarly, according to the standard test practice, each testing procedure was repeated 12 times, and the results demonstrated below refer to the mean over these runs. Table 5 summarizes the properties of the signals acquired during the pilot trials, such as the number of available signals, how these samples are distributed to each subset for the training, validation, and testing of our methodology, and general properties characterizing each time series.

**Figure 10:**
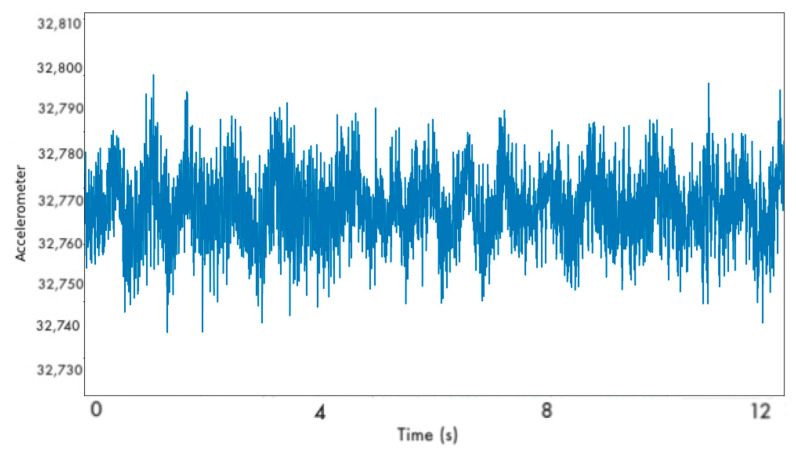
Reconstruction Error of the LSTM autoencoder.

The detection scheme follows the same pattern as described for the experimental testing. First, the LSTM AE model is responsible for the identification of leakages by detecting abnormalities in the signal. Second, after the LSTM AE has detected potential failure, the procedure of creating spectrograms with a 20-s rolling window is initiated Figure 10). These spectrograms are then received by a 2D-CNN which undertakes the classification of the operational state of the monitored pipeline. Table 5 lists the selected architecture and hyperparameter configuration of the models composing the monitoring scheme in the actual operating environment.

**Figure 11:**
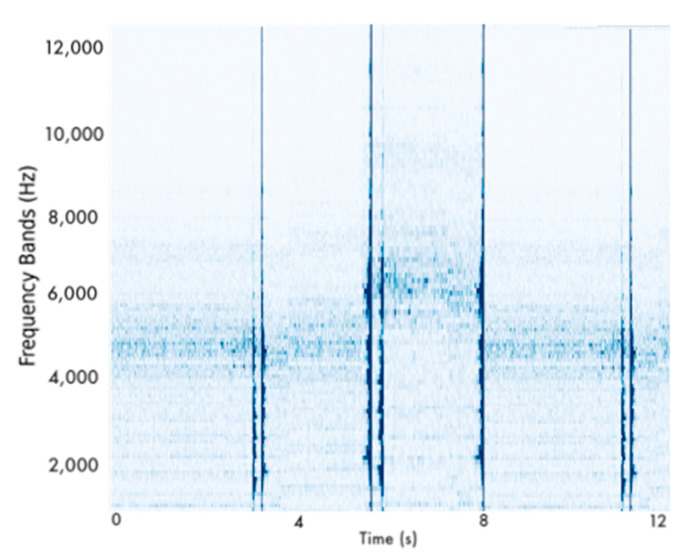
Example of a defective spectrogram extracted from Kalochori dataset.

Subsequently, the generation of the spectrograms begins. Indicatively, Figure 11 displays an example of such a spectrogram. The detection of the leakage is considered successfully identified by our monitoring scheme when it is correctly and timely recognized by the LSTM AE, and the 2D-CNN continues flagging the signal as defective. In case one of these conditions is not satisfied, then the observation is deemed as misclassified.

**Figure 12:**
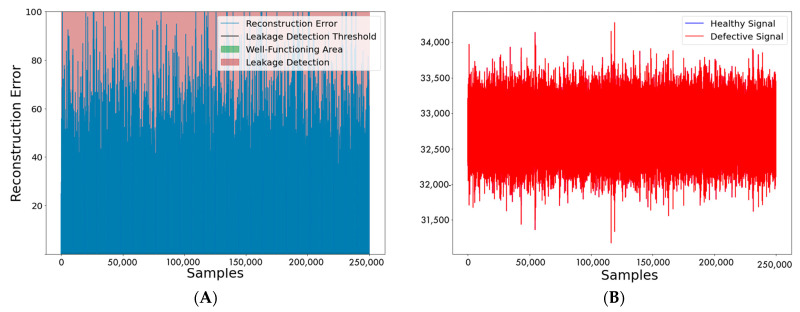
(**A**): The acoustic signal. With blue, the healthy signal is represented, and with red the signal after the leakage. (**B**): Reconstruction Error of the LSTM autoencoder.

Lastly, the efficacy of these models in detecting outflow is presented. Indicatively, Figure 12 demonstrates the LSTM autoencoder while inspecting a healthy and defective signal. Figure 12 (right) demonstrates the reconstruction error, and in Figure 12 (left), the actual acoustic signal acquired from the monitored pipeline is represented. The red denotes the outflow, as it can also be seen that no blue portion can be discerned because the outflow began before the monitoring.

The proposed methodology was again evaluated under diverse circumstances regarding the distance of the leakage from the nodes, the circulation of the fluid, the leakage diameter to determine the effect of the rupture’s size, and the distance from the nodes on the efficacy of the models. In this round of experiments, due to limitations in varying the leakage diameter, the effect of the distance from the node was further audited. Table 6 summarizes the performance of the model in terms of accuracy detection, for each of the aforementioned instances, along with the results yielded by our previous studies concerning the components of our combined approach to the task of anomaly detection in the piping networks.

Furthermore, diverse metrics were used to obtain a more comprehensive understanding of the different models’ performance and enabled us to define the deficiencies of the model better and whether they are more susceptible to False Positives or False Negatives. The metrics employed are as follows:

(13) $$Accuracy=\frac{TP+TN}{TP+FP+TN+FN},\;$$

(14) $$Precision=\frac{TP}{TP+FP},\;$$

(15) $$Recall=\frac{TP}{TP+FN},\;$$

(16) $$Specificity=\frac{TN}{TN+FP},\;$$

where *TP*, *TN*, *FP*, *FN* denotes the True Positive, True Negative, False Positive, and False Negative, respectively.

As demonstrated in Table 6, solely regarding the proposed methodology, highly accurate results were yielded even in an actual operating environment with substantial external noise. It was observed that as the distance from the node increased, the accuracy of our methodology decreased, maintaining very high accuracy yields across the numerous trials. Most importantly, it was also evinced that the propounded combined method presented in this study considerably improved the accuracy of detecting anomalies in the signal of the pipelines. Additionally, it is presented that the individual components were more susceptible to different types of errors. More specifically, the LSTM AEs were prone to label a signal erroneously as healthy, as demonstrated by the relatively lower recall values. This phenomenon is explained by the fact that it was commonly observed that, especially on the occasion of leakages with a small diameter, the signal resembled the signal before the occurrence of the leakage, thus misleading the LSTM AE model. Furthermore, the CNN classifiers presented a more balanced performance while slightly tilted towards falsely detecting leakages in healthy samples. Hence, it is further illustrated how the combined approach is capable of merging the two components and yielding better performance.

Lastly, despite having established the efficacy of the propounded methodology in the experimental as well as in the actual pipeline setup, it is intrinsic to compare our recommended models to other algorithms widely employed in pertinent literature for the task of anomaly detection. From our previous study, it was deduced that the AutoRegressive Moving Average for modeling univariate stationary time series performed best out of the set of benchmark models. Therefore, it is selected to provide a benchmark for comparison with the results obtained by the combined approach. The ARMA model is similarly trained solely using the dataset from the experimental pipeline network in Kalochori. This method is based on a regression model that is first fitted to the training data. Then the resulting model is used to forecast test sequences, and the difference between the predicted and real values is called residual. Suppose the orders p and q of the AR and MA models, respectively, have been chosen appropriately to model the given time series. In that case, it follows that the residuals are assumed to be distributed normally. Subsequently, these residuals are utilized to calculate the rolling z-score of the prediction error. Assuming that the fitted model is capable of satisfactorily predicting the healthy time series provided in the training step, if the error continuously exceeded the 95% confidence interval, it would serve as an anomaly indicator since the model would fail to predict the time series accurately based on the system dynamics learned during training signifying a significant change in the system. The input time series were ascertained to be stationary through the Augmented Dickey–Fuller test for our problem. The AR and MA order found from the training of the ARMA models to adequately capture the piping system’s dynamics were p = 4 and q = 5.

As Table 7 reveals, there is a significant performance gap between the presented approach and the ARMA models. More specifically, the ARMA model struggles to maintain high levels of accuracy. This should likely be accredited to the fact that the ARMA model was trained based on the dataset of the experimental setup and was then asked to generate forecasts to the signals from the oil refinery. Conversely, it is evinced that the posed combined methodology demonstrates significantly greater transferability, allowing the model stored on the edge to perform real-time leakage detection, despite being trained on the experimental pipeline setup.

## 6. Conclusions

During recent decades, the ever-growing oil industry has highlighted the importance of supervising the integrity and efficient operation of piping systems worldwide. Monitoring the pipeline network operational condition and the timely detection of malfunctions of energy systems contribute to the minimization of environmental, economic, and social consequences. The critical challenge is the timely and accurate data acquisition from sensors integrated into pipelines set in industrial and harsh environments. The methodologies are part of the ESTHISIS project, which aims to detect leakages in oil and gas pipelines by gathering and processing data from accelerometers placed alongside the pipelines, forming an edge computing system that can issue early warning notifications on leakages.

The focal point of the present study is to establish a new combined methodology for leakage detection, and based on the data acquired from an intelligent wireless system for leakage detection in pipelines for oil and gas transportation and storage has been developed. More specifically, the signal from the pipelines is constantly fed to the LSTM AE, which undertakes the task of detecting anomalies instantaneously. Subsequently, based on our failure decision process, the operational state of the pipeline is either labeled as healthy or defective. Lastly, on the occasion of a defective pipeline, the signals thereafter are converted into spectrograms which are subsequently fed to CNN classifiers to achieve continuous flagging of the state as defective. Two separate trials took place in two distinct settings. First, the experimental setup in Kalochori was utilized for the training of the models, which would be subsequently used in the testing environment. The second implementation concerned an actual operating environment in an oil refinery. The main challenge in this setup was the considerable ambient noise extant in the measurements, instigating external noise entering the monitored system, which could potentially decrease the detection accuracy of our models.

However, it was demonstrated that the combined methodology managed to bridge the two components harmoniously and successfully conceal their respective weaknesses, as these models achieved near-perfect or, on some occasions, even perfect classification accuracy for the leakage detection task on the signals stemming from the oil refinery. More specifically, the LSTM AE contributes to the instantaneous and timely detection of leakages when they occur; nonetheless, in [EAAI], it was demonstrated that they were susceptible to false negatives, as the signal from the pipeline wall resembled noise for small leakages. This deficiency is compensated with the 2D-CNN classifiers, which were employed in classifying spectrograms in which the time point when the leakage occurred was purposefully omitted. Additionally, this approach offers the alternative of storing lengthy signals to store static low-resolution images that occupy considerably less memory.

The primary innovation of the presented integrated system concerns accurate and timely leakage detection. The system can contribute to preventing possible environmental disasters and incidents in the fuel industry and the future evolution of intelligent sensor solutions for liquid and gas storing and transporting procedures. Additionally, to the best of the authors’ knowledge, this is the first study implementing 2D-CNNs classifiers receiving spectrograms for the detection of leakage. Moreover, not only did we demonstrate the applicability of this combination of NN genres but also, we successfully demonstrated that this monitoring scheme could identify changes in the vibrations in the pipeline system different than the one that was used for its training. Furthermore, the neural networks presented in this study were also compared with the individual network components from our previous study, and ARMA models were used as a performance benchmark. The results demonstrated that the combined model did outperform the benchmark model, being more accurate overall, but it also outperformed its components.

## Figures and Tables

**Table 1:** ESTHISIS Platform Technical Specifications.

**Frequency Range**	0.5 to 25 kHz (User-Defined)
**Number of channels**	4
**Resolution**	16 bits
**GNSS**	BeiDou, Galileo, GLONASS, GPS/QZSS
**time pulse signal**	30 nsec (RMS), 60 nsec (99%)
**MCU Operating frequency**	250 Mhz
**MCU Integrated PSRAM**	8 MB
**CPU**	Dual-core Cortex-A72 up to 1.8 GHz
	Quad-core Cortex-A53 up to 1.4 GHz
**CPU RAM**	3 GB LPDRR3 (CPU 2 GB + NPU 1 GB)
**CPU Flash**	16 GB eMMC

**Table 2:** Dataset properties.

Dataset Properties	Value
No leak signals (train-validation-test)	120 (80-15-25)
Leak signals (train-validation-test)	120 (80-15-25)
Inspection time per signal	10 s
Sampling frequency (Hz)	25 kHz
Signal length	250,000-time steps
Leakage Diameter (mm)	1–7
Node Distance (cm)	1810, 2260, 3530

**Table 3:** Hyperparameter selection—Long Short-Term Memory AutoEncoder.

Leakage Diameter (mm)	Value
LSTM layer 1 units (Encoding 1st—Decoding 2nd)	128
LSTM layer 2 units (Encoding 2nd—Decoding 1st)	64
Learning rate	2 × 10^−4^
““Lookback”” window	5
Epochs	200
Batch size	8

**Table 4:** Hyperparameter selection—Convolutional Neural Network.

Leakage Diameter (mm)	Value
Convolutional Layer #1	256 × 256, Kernels: 3 × 3
Convolutional Layer #2	32 × 32, Kernels: 3 × 3
Max Pooling Layer #1	32 × 32, Kernels: 2 × 2
Convolutional Layer #3	32 × 32, Kernels: 3 × 3
Max Pooling Layer #2	32 × 32, Kernels: 2 × 2
FCN Layer #1	15 nodes, Dropout = 0.3
Output Layer	2 nodes
Learning Rate	5 × 10^−4^
Weight updates	Epochs × BatchSize = 8 × 100 = 800

**Table 5:** Dataset properties.

Dataset Properties	Value
No leak signals	103 (70-10-23)
Leak signals	97 (70-7-20)
Inspection time per signal	10 s
Sampling frequency (Hz)	25 kHz
Signal length	250,000-time steps
Leakage Diameter	5 mm, 13 mm
Node Distance	850, 1350, 2260, 2820,3350

**Table 6:** Leakage detection performance from oil refinery trials of the (a) proposed combined model, (b) LSTM AE, (c) CNN Classifier.

**Leakage (mm)**	**Node Distance (cm)**	**Combined Accuracy (%)**	**LSTM AE-Accuracy (%)**	**CNN-Accuracy (%)**
5 mm	850	100	93.0	96.1
13 mm	850	100	96.4	99.0
5 mm	1350	99.5	92.1	94.2
13 mm	1350	100	94.9	96.6
5 mm	2260	97.9	91.5	90.7
13 mm	2260	99.3	92.0	92.9
5 mm	2820	96.7	86.3	87.4
13 mm	2820	99.0	88.8	90.2
5 mm	3350	96.7	81.8	83.9
13 mm	3350	98.2	84.7	88.3
**Leakage (mm)**	**Node Distance (cm)**	**Combined Precision (%)**	**LSTM AE-Precision (%)**	**CNN-Precision (%)**
5 mm	850	100	92.0	91.3
13 mm	850	100	95.8	93.9
5 mm	1350	99.3	89.6	88.7
13 mm	1350	100	92.4	91.6
5 mm	2260	98.3	85.1	87.1
13 mm	2260	99.0	88.8	90.5
5 mm	2820	96.2	83.8	84.9
13 mm	2820	98.2	86.7	88.1
5 mm	3350	97.0	82.0	83.4
13 mm	3350	98.0	85.2	85.2
**Leakage (mm)**	**Node Distance (cm)**	**Combined Recall (%)**	**LSTM AE-Recall (%)**	**CNN-Recall (%)**
5 mm	850	100	90.9	93.1
13 mm	850	100	92.0	95.9
5 mm	1350	99.2	88.0	90.7
13 mm	1350	100	90.3	93.4
5 mm	2260	97.1	84.3	90.1
13 mm	2260	99.3	87.1	92.4
5 mm	2820	96.5	82.9	88.3
13 mm	2820	98.0	85.6	90.6
5 mm	3350	96.4	78.2	87.2
13 mm	3350	98.4	81.5	89.5
**Leakage (mm)**	**Node Distance (cm)**	**Combined Specificity (%)**	**LSTM AE-Specificity (%)**	**CNN-Specificity (%)**
5 mm	850	100	93.5	93.0
13 mm	850	100	95.3	95.9
5 mm	1350	99.5	88.3	89.7
13 mm	1350	100	92.7	92.6
5 mm	2260	97.9	84.0	88.1
13 mm	2260	99.3	89.2	90.5
5 mm	2820	96.7	83.4	87.9
13 mm	2820	99.0	86.1	89.1
5 mm	3350	96.7	81.3	83.4
13 mm	3350	98.2	84.8	86.2

**Table 7:** Leakage detection performance from oil refinery trials of the (a) proposed combined model, (b) ARMA model.

**Leakage (mm)**	**Node Distance (cm)**	**Combined Accuracy (%)**	**ARMA Accuracy (%)**
5 mm	850	100	88.3
13 mm	850	100	90.2
5 mm	1350	99.5	86.2
13 mm	1350	100	89.1
5 mm	2260	97.9	80.5
13 mm	2260	99.3	84.4
5 mm	2820	96.7	77.3
13 mm	2820	99.0	81.9
5 mm	3350	96.7	75.0
13 mm	3350	98.2	79.6
**Leakage (mm)**	**Node Distance (cm)**	**Combined Precision (%)**	**ARMA** **-Precision (%)**
5 mm	850	100	85.2
13 mm	850	100	88.7
5 mm	1350	99.3	87.0
13 mm	1350	100	87.9
5 mm	2260	98.3	83.9
13 mm	2260	99.0	85.6
5 mm	2820	96.2	78.4
13 mm	2820	98.2	81.3
5 mm	3350	97.0	76.8
13 mm	3350	98.0	79.8
**Leakage (mm)**	**Node Distance (cm)**	**Combined Recall (%)**	**ARMA**-**Recall (%)**
5 mm	850	100	84.9
13 mm	850	100	87.3
5 mm	1350	99.2	84.2
13 mm	1350	100	85.8
5 mm	2260	97.1	81.9
13 mm	2260	99.3	84.4
5 mm	2820	96.5	79.5
13 mm	2820	98.0	80.9
5 mm	3350	96.4	74.9
13 mm	3350	98.4	78.7
**Leakage (mm)**	**Node Distance (cm)**	**Combined Specificity (%)**	**ARMA**-**Specificity (%)**
5 mm	850	100	89.8
13 mm	850	100	90.3
5 mm	1350	99.5	86.2
13 mm	1350	100	87.8
5 mm	2260	97.9	82.9
13 mm	2260	99.3	84.4
5 mm	2820	96.7	79.5
13 mm	2820	99.0	80.9
5 mm	3350	96.7	74.3
13 mm	3350	98.2	79.8
